# Big Five Personality, Academic Entrepreneurial Motivation, and Academic Entrepreneurial Intention: A Research Method Based on Fuzzy Set Qualitative Comparative Analysis

**DOI:** 10.3389/fpsyg.2021.799770

**Published:** 2022-04-20

**Authors:** Yuying Zhang, Peng Wang, Yanzhi Zhao

**Affiliations:** ^1^School of Public Administration, Dongbei University of Finance and Economics, Dalian, China; ^2^Institute of China Innovation and Entrepreneurship Education, Wenzhou Medical University, Wenzhou, China

**Keywords:** academic entrepreneurial intention, configuration thinking, fsQCA, Big Five personality, academic entrepreneurial motivation

## Abstract

Scholars are the main force behind academic entrepreneurship. The method of how to stimulate scholars’ academic entrepreneurial intention and how to further promote social and economic development are important questions for the academic community. Research on the “net effect” of the factors affecting academic entrepreneurial intention has achieved some theoretical results. However, the results that affect academic entrepreneurial intention are complex and not influenced by a single factor, but rather by the interaction between various factors. Therefore, this study used a fuzzy set qualitative comparative analysis research method to explore how various factors can affect scholars’ academic entrepreneurial intention from two dimensions: the Big Five personality traits and academic entrepreneurial motivation. Our findings showed two configurations that affect high academic entrepreneurial intention of university scholars: the openness to experience—ribbon—dominant path, and the ribbon—dominant path. Additionally, two configurations were revealed for the formation of not-high academic entrepreneurial intention: extraversion—conscientiousness—inhibition and extraversion—agreeableness—gold—hindrance paths. Moreover, this study revealed that a causal asymmetry exists between the high and the not-high academic entrepreneurial intention configurations. This study broadens the application of the fuzzy set qualitative comparative analysis method in the research of academic entrepreneurial intention and provides theoretical and practical insights for researchers and practitioners on how to effectively stimulate scholars’ academic entrepreneurial intention.

## Introduction

Academic entrepreneurship is the key to promoting high-quality economic development and is an important component of the implementation of the national innovation system (Schmitz et al., 2017). In a narrow sense, academic entrepreneurship refers to commercialized entrepreneurial activities carried out by individual scholars or academic organizations based on scientific research results (Louis et al., 1989; Klofsten and Jones-Evans, 2000). Its most common forms include licensing, technology transfer, and spin-offs (Wang et al., 2021a). Academic entrepreneurship plays an increasingly prominent role in realizing the academic research achievements of universities and serving society as the commercial mission of universities. Therefore, in the era of the knowledge economy, stimulating the academic entrepreneurial willingness of university teachers and improving their academic entrepreneurial enthusiasm has been a concern for scholars.

Academic entrepreneurial intention reflects the attention and behavior of academic entrepreneurs and leads to a subjective psychological state of determining whether they are willing to establish spin-offs, licensing, technology transfer, or similar activities. Previous studies have extensively discussed the factors that affect university teachers’ academic entrepreneurial intention including personal factors, such as personality traits (Kolb and Wagner, 2015; Cantner et al., 2017; Obschonka et al., 2019; Vega-Gomez et al., 2020) and job satisfaction (Blaese et al., 2021); financial factors, such as scholars’ scientific research (which needs further financial support) (Foo et al., 2016), or improving personal economic interests (Goethner et al., 2012; Vega-Gomez et al., 2018); academic factors, such as seeking an identity as an expert among colleagues in the academic community (Obschonka et al., 2012), being supported by excellent leaders (Johnson et al., 2017), or being in a good academic entrepreneurial environment and atmosphere of entrepreneurial culture among academic groups (Huyghe and Knockaert, 2015; Wang et al., 2021b); and social support factors, such as scholars’ parents owning an enterprise, finding an entrepreneurial role model in peers (Moog et al., 2015), or commercialization attitude and social support (Acuna-Duran et al., 2021; Wang et al., 2021b). These factors affect scholars’ academic entrepreneurial intention to varying degrees. However, existing research still needs further supplementation. First, the influence of the “interrelationship” of various factors on the results has been ignored. Throughout existing literature, the impact of net effect of factors on scholars’ academic entrepreneurial intention has been the main focus for analysis. However, from a practical point of view, academic entrepreneurial intention is not directly affected by several factors, but by the complex interaction of many factors. Second, the influence of the combination of multiple paths on the results has been disregarded. Results are produced by the combination of many different factors, rather than on a “one-to-one” factor basis. Third, existing literature has failed to recognize a large number of asymmetric realities. Previous studies based on traditional regression analysis or structural equation model mainly analyzed problems with the idea of linearity and symmetry, ignoring the analysis of a large number of asymmetric facts.

The fuzzy set qualitative comparative analysis (fsQCA) research method replaces net effect thinking with configuration thinking, which can better address the complexity of causality and the above mentioned research limitations. Hence, this study uses the fsQCA research method, based on personality trait theory and self-determination theory, to analyze how the combination of two dimensions (i.e., eight condition variables) of multiple motivation and personality traits can better promote the high (or not-high) academic entrepreneurial intention of university teachers from the perspective of configuration, and help scholars and practitioners better understand the driving or inhibiting factors.

Three key questions shall be examined in this study: What are the core condition variables that affect high or not-high academic entrepreneurial intention? Which paths can better promote scholars’ academic entrepreneurial intention? Which paths will inhibit scholars’ academic entrepreneurial intention?

The structure of this study are as follows. The second section is a literature review about the Big Five personality and academic entrepreneurial motivation. The third section describes the research methods of fsQCA, samples, and scale sources. The fourth part reports the results of this study. It includes the configuration that produces high (or not-high) academic entrepreneurial intention, and a robustness analysis is carried out to ensure the accuracy of the results. The fifth part discusses and summarizes this study, defines the theoretical significance, practical significance, and limitations of this study, and puts forward suggestions for future research directions.

## Literature Review and Research Framework

Based on personality trait theory and self-determination theory, this study explores the configuration effects of different conditional variables on scholars’ academic entrepreneurial intention. Previous studies have mainly focused on specific entrepreneurial personality traits, such as adventure, innovation, and achievement needs. Since the 1980s, the Big Five personality model has been gradually recognized by scholars as the main reference system for studying entrepreneurial personality traits (Zhao and Seibert, 2006; Sahin et al., 2019). While scholars have identified a positive correlation between general extraversion, conscientiousness, emotional stability, and openness to experience in the Big Five personality model, there are still different opinions on whether agreeableness has a positive impact on entrepreneurial intention. These studies have deeply investigated the relationship between entrepreneurial personality traits and entrepreneurial intention. However, we believe it is insufficient to only consider the impact of entrepreneurial personality traits on entrepreneurial intention, because entrepreneurial motivation is also an important factor driving entrepreneurial activities. Personality traits affect entrepreneurial motivation, and different personality traits will in turn affect and stimulate different entrepreneurial motivation and intention. Self-determination theory points out that an individual’s decision to participate in an activity is influenced by individual needs or external incentives. Based on this theory, Lam (2011) further summarized three motives that affect scholars’ academic entrepreneurship, which have good recognition and authority. Therefore, the literature review for this study was carried out from two dimensions: the Big Five personality and academic entrepreneurial motivation.

### Big Five Personality and Academic Entrepreneurial Intention

Big Five personality (extraversion, agreeableness, conscientiousness, emotional stability, and openness to experience) is a psychological research model that focuses on a certain tendency reflected by individual psychological characteristics. It is not only a decisive factor for entrepreneurial intention, but also a key variable affecting entrepreneurial intention (Sahin et al., 2019). Extraversion refers to a trait in which individuals are energetic, independent, outgoing, enthusiastic, and can actively express themselves (McCrae and Costa, 1992). Entrepreneurial activities require entrepreneurs to have more social interactions with people. Therefore, extroverted entrepreneurs generally show self-confidence, enthusiasm, and excellent social skills in social occasions, and can occupy a dominant position in the entrepreneurial process (Zhao et al., 2010). Kolb and Wagner (2015) believed that scholars engaged in entrepreneurship, like other entrepreneurs, showed a higher level of extraversion in the process of entrepreneurship. Wang et al. (2016) pointed out that extraversion positively affects external network entrepreneurial motivation, and promotes entrepreneurial intention. Yeh et al.’s (2020) research is consistent with the results of Wang et al. (2016). Both believed that extroversion can predict external motivation well and have a positive impact on entrepreneurial intention. Although there is a positive correlation between extraversion and entrepreneurial intention, other scholars believe that extraversion has no significant influence on entrepreneurs (Hmieleski and Corbett, 2006). Cantner et al. (2017) found through a questionnaire survey of German scientists that, especially in the case of university scholars, extraversion had no significant impact on their academic entrepreneurial intention. A possible reason was that the solidification of their role made scholars feel more introverted, because they spent more time working for themselves in a fixed range, to further improve and gain insights in academic research (Feist, 1998; Kolb and Wagner, 2015).

Agreeableness mainly refers to the positive relationship between tolerance, selflessness, trust, and altruism toward others (Ciavarella et al., 2004; Obschonka et al., 2019). For entrepreneurs, such characteristics can help them maintain and develop cooperative relationships, especially for the long-term development of new enterprises (Ciavarella et al., 2004). However, Zhao and Seibert (2006) found that entrepreneurs scored lower than managers in terms of agreeableness. Extremely high levels of agreeableness reflect humility, and the fact that it is easy to be deceived by others in interpersonal relationships which is not conducive to becoming qualified entrepreneurs. Zhao and Seibert (2006) believed that the personality gap between entrepreneurs and non-entrepreneurs could be verified from attraction-selection-attrition (ASA) theory in the future. Therefore, Zhao et al. (2010) further expanded their research, and the results showed that agreeableness had no significant impact on entrepreneurial intention. However, existing literature shows that the impact of agreeableness on scholars’ academic entrepreneurial intention is twofold. Kolb and Wagner (2015) believed that entrepreneurs with a university background had a higher level of agreeableness in the process of entrepreneurship, because scholars preferred to show their good academic reputation in the process of cooperation in order to maintain a good cooperative relationship and communicate with others in the process of academic entrepreneurship (Barrick et al., 2003). But, the results of Cantner et al. (2017) were different. They believed that, especially for university scholars, agreeableness had no significant impact on their academic entrepreneurial intention. Hoyte (2019) is consistent with that of Cantner et al. (2017), as they concurred that agreeableness was the only personality trait that had no significant impact on entrepreneurial intention. Vega-Gomez et al. (2020) also reached the same research conclusion. They too believed that agreeableness had no impact on scholars’ entrepreneurship, and the possible reason was that agreeableness is a personality trait shared by entrepreneurs and non-entrepreneurs alike.

Conscientiousness refers to an individual’s reliability in the completion of tasks and goals, showing persistence, reliability, and responsibility (Presenza et al., 2020). Entrepreneurs with good conscientiousness think first and then act on goals and tasks, and can carry out entrepreneurial activities in a planned and organized manner. Zhao and Seibert (2006); Zhao et al. (2010) showed that entrepreneurs with strong sense of conscientiousness stimulated more entrepreneurial intention. Over time, more scholars have verified the positive role of entrepreneurs’ conscientiousness in entrepreneurial intention. For example, Brandstaetter (2011) found that conscientiousness positively affected entrepreneurial intention and entrepreneurial performance, and the score was higher for entrepreneurs than that for non-entrepreneurs. Wang et al. (2016) found that conscientiousness positively affected internal and external entrepreneurial motivation and entrepreneurial intention based on a structural equation model. Similarly, if scholars show high conscientiousness, they will have more academic research achievements and will expect their scientific research to be more significant, such as serving society and improving the quality of economic development. Cantner et al. (2017); Vega-Gomez et al. (2020) both believed that scholars’ entrepreneurial intention and conscientiousness were positively related. Their research indicated that scholars with a high level of conscientiousness showed higher courage in academic entrepreneurial activities. Therefore, scholars with good conscientiousness will show more entrepreneurial spirit and actively participate in academic entrepreneurial activities to achieve their purpose of applying academic research results to practice (Obschonka et al., 2019).

Emotional stability is a reverse measure of neuroticism. Neuroticism refers to the tendency of individuals to easily experience negative emotions, such as hostility, tension, depression, and anxiety. Emotional stability refers to individuals’ tendency to experience positive emotions, such as peace, relaxation, strength, calmness, and self-confidence (Vega-Gomez et al., 2020). Zhao and Seibert (2006) revealed that entrepreneurs scored higher than non-entrepreneurs in emotional stability. Subsequently, Rauch and Frese (2007)’s meta-analysis also obtained similar results, showing that good emotional stability helped individuals maintain good interpersonal relationships in entrepreneurial activities. With regards to the academic entrepreneurship of scholars, other scholars have also conducted relevant research. For example, Kolb and Wagner (2015) ascertained that there was no significant difference in the emotional stability between entrepreneurs in an academic environment and entrepreneurs outside the university domain. Cantner et al. (2017) found similar results through a regression analysis; that is, emotional stability had no significant correlation with scholars’ academic entrepreneurial intention. However, Vega-Gomez et al. (2020) conducted a questionnaire survey of 799 Spanish scholars using Partial Least Squares (PLS) regression technique and found that a high level of openness, extroversion, and emotional stability were important antecedents of scholars’ entrepreneurial skills or entrepreneurial intention. In particular, scholars with high emotional stability can adapt themselves in the face of heavy scientific research pressure, maintain positive emotions, and transform scientific research achievements into commercial achievements so that their research is conducive to social development (Van Ness and Seifert, 2016).

Openness to experience refers to an individual’s curiosity in intelligence, innovative ideas, and creativity in thinking (Antoncic et al., 2015). Individuals with high openness to experience are more able to have unique entrepreneurial creativity and entrepreneurial spirit, and are more willing to experience new things. In the face of entrepreneurial difficulties, being able to use creative thinking to solve problems is a crucial factor in distinguishing entrepreneurs from ordinary people (Schmitt-Rodermund, 2004). Zhao and Seibert (2006) and Zhao et al. (2010) pointed out that the most distinguishing personality characteristic of entrepreneurs from non-entrepreneurs is that entrepreneurs have a high level of openness to experience. Consistent with this finding, Wang et al. (2016); Yeh et al. (2020) believed that a high level of openness to experience positively affects internal entrepreneurial motivation, thus affecting entrepreneurial intention. The research method adopted by Sahin et al. (2019) was different from previous studies. Sahin et al. (2019) verified that openness to experience plays a central role in all configurations based on the fsQCA method, and that openness to experience positively affected entrepreneurial intention. However, according to Kolb and Wagner (2015), scholars have lower levels of openness to experience, the possible reason being that although university scholars may face the uncertainty of entrepreneurship, but the future is predictable. Therefore, scholars prefer to pursue a stable and reliable environment. Hence, Kolb and Wagner (2015) believed that entrepreneurs should have a high level of extraversion, conscientiousness, emotional stability, and openness to experience, and low level of agreeableness, which is not applicable to all types of entrepreneurs (e.g., including student entrepreneurs, academic entrepreneurs, or social entrepreneurs). Yet, in contrast to the research of Kolb and Wagner (2015); Cantner et al. (2017) found that openness to experience positively affected scholars’ academic entrepreneurship intention. Scholars in colleges and universities were more innovative and more willing to apply their creative research results to entrepreneurship, so as to benefit more people. Furthermore, Vega-Gomez et al. (2020) believed that scholars’ academic entrepreneurship highlighted a high level of openness to experience, because academic research reflects creativity. It involves innovation based on previous research, such as discovering new research perspectives, theories, contents, and methods. Scholars with a high degree of openness to experience are more able to show academic entrepreneurial intention because they are more willing to apply new knowledge research content to the development of goods or services.

### Academic Entrepreneurial Motivation and Academic Entrepreneurial Intention

As a mature and widely studied concept, motivation is the endogenous driving force that moves people to act (Wang et al., 2021d). Academic entrepreneurial motivation refers to the driving factors that promote scholars to carry out academic entrepreneurial activities and involves the intensity and sustainability of goal-oriented behavior. In existing research, there are many classifications of academic entrepreneurial motivation, usually based on the nature of academic entrepreneurship. The generally accepted classifications are extrinsic and intrinsic motivation, and monetary and non-monetary motivation (D’Este and Perkmann, 2011; Nsanzumuhire and Groot, 2020). Lam (2011) summarized academic entrepreneurial motivation under the classifications “ribbon” (i.e., reputational/career rewards), “puzzle” (i.e., intrinsic satisfaction), and “gold” (i.e., financial rewards). Therefore, this study specifically analyzed the impact of these three types of motivation on academic entrepreneurial intention.

Ribbon motivation (i.e., reputation or career rewards motivation) is not only an external motivation but also a motivation for scientific research. Scientific reputation and model support (Johnson et al., 2017) directly affect scholars’ academic entrepreneurship activities. Merton (1957) “priority theory” provides a theoretical basis for scholars to publish academic works in public in order to obtain the recognition of their peers. Scholars are more willing to disclose the research progress of knowledge to the outside world through patents, licenses, and public publications, in order to obtain the recognition of academic peers and improve their academic reputation and status in the academic community (Meoli and Vismara, 2016; Hayter and Feeney, 2017; Wang et al., 2021a). Therefore, this type of academic entrepreneurial motivation is an external motivation to further obtain professional promotion and reward. In contrast to entrepreneurs in the general sense, academic entrepreneurs emphasize the commercialization of scientific research achievements. Therefore, academic entrepreneurs aim to achieve their research objectives and apply results to practice in order to make meaningful academic contributions to their research field and promote the development of society. Iorio et al. (2017) emphasized that this is a kind of prosocial motivation, which aims to expand the research mission of universities, promote regional and social development, and satisfy the desire to be useful to society. Chiesa and Piccaluga (2000); Uctu and Jafta (2012) jointly pointed out that scholars’ motivation to establish derivative enterprises is to apply their results to practice. In addition, van de Burgwal et al. (2019) believed that ribbon motivation is a kind of professional motivation, which involves the ability of scholars to provide employment opportunities for peers or students.

Puzzle motivation (i.e., intrinsic motivation) is also known as personal motivation. Academic circles agree that it is an important driving factor that promotes scholars’ willingness to engage in academic entrepreneurship. Academic entrepreneurship activities can not only open up new research fields, but also help meet scholars’ internal challenges and difficulties. In satisfying intellectual curiosity or contributing to technology, academic entrepreneurs obtain internal satisfaction and enhance their self-worth (Balven et al., 2018). Blind et al. (2018), based on the motivation research framework of Lam (2011), found that German scholars’ standardization was more motivated by internal motivation, with the most fundamental purpose being to solve problems and obtain internal interest. van de Burgwal et al. (2019) reached the same conclusion. Thus, this kind of motivation comes more from an individual’s need to pursue academic success and achievement. Scholars are more willing to obtain internal satisfaction from personal success when solving challenging activities or exploring new fields of research, rather than focusing on the maximization of short-term profits.

Gold motivation (i.e., financial rewards motivation) includes bonuses, salary increases, transfer fees, and royalties for the transfer of knowledge and technology (Sung and Gibson, 2005). There are still many disputes about whether financial rewards motivation is in fact the main motivation to encourage scholars to start academic entrepreneurship. Some scholars believe that financial motivation indirectly impacts entrepreneurial intention (Goethner et al., 2012). Perkmann et al. (2013) believed that scholars engaged in entrepreneurial activities are usually motivated by financial interest. As some scholars are paid less to better improve their living conditions, they are more willing to participate in entrepreneurial activities in pursuit of higher private income and returns (D’Este and Perkmann, 2011). This is especially true for senior scholars who have proven that they carry out academic entrepreneurship activities out of the motivation for further financial support (Rizzo, 2015; Janger and Nowotny, 2016). However, Hayter (2011) held a different opinion. Based on an in-depth interview with 74 nascent academic entrepreneurs, he found that scholars’ motivation for academic entrepreneurship is multifaceted, and includes elements such as becoming a role model among peers, applying results to public services, and enriching scholars’ professional life. Therefore, monetary motivation does not seem to have a high impact on scholars. The limitation of Hayter (2011) study was that it ignored the role of time. Therefore, in order to make up for this limitation, Hayter (2015) further interviewed 57 academic entrepreneurs and found that scholars’ academic entrepreneurial motivation changed over time, paying more attention to providing employment opportunities for students or employees and avoiding the bureaucratic learning atmosphere of the university. Therefore, monetary motivation had less of an effect. Money is a small incentive, or even unimportant, because the fundamental purpose of scholars’ academic entrepreneurial activities is to engage in higher academic pursuits; thus financial motivation is not the starting point (van de Burgwal et al., 2019).

### Research Framework

**FIGURE 1:**
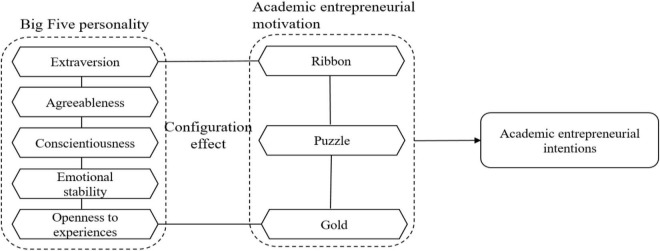
Research framework.

Based on the above literature, academic entrepreneurial motivation and the Big Five personality traits provide a selection basis for the selection of condition variables in this study. There is no research on the complex interaction between various motivational elements and the synergy of scholars’ academic entrepreneurship. Therefore, this study constructed a research framework based on how the eight condition variables of the Big Five personality traits (extraversion, agreeableness, conscientiousness, emotional stability, and openness to experience) and academic entrepreneurial motivation (ribbon, puzzle, and gold) can better promote or inhibit academic entrepreneurial intention (see Figure 1).

## Research Methods and Data

### Research Methods

The fsQCA is one of the methods of qualitative comparative analysis (QCA). With the idea of set theory, we can analyze research problems that change in level or degree, and can handle values between 0 and 1 after calibration. The fsQCA method combines the advantages of qualitative analysis and quantitative analysis, takes the specific combination of conditional variables of the research problem as the sufficient condition of the result, and analyzes the influence of the complex configuration between conditional variables on the outcome variables from the perspective of whole and configuration cognition thinking (Ragin, 2008). The fsQCA method has gradually attracted the attention of scholars and is widely used in business (Pappas et al., 2016), management (Kraus et al., 2018), psychology (Thai and Wang, 2020), and other disciplines. The fsQCA method has been adopted in this study for the following reasons: first, with respect to the QCA method, the fsQCA method has more advantages in analyzing continuous variables^1^ than the crisp set qualitative comparative analysis (csQCA^2^) method and multi-value qualitative comparative analysis (mvQCA) method. Second, in contrast to the traditional regression analysis method (i.e., exploring the ‘‘net effect’’ analysis of factors and ignoring the interdependence between independent variables), the fsQCA method can reveal the influence of the complex configuration relationship of multiple factors on the results, and the non-conflicting configuration equivalence of the combination of multiple antecedent conditions on the outcome variable (e.g., in this study, four configurations are found, which explain the high or not-high academic entrepreneurial intention to varying degrees). Third, the advantage of the fsQCA method is that it is also suitable for both large sample research as well as small and medium-sized sample research, breaking through the limitations of econometric and statistical methods such as regression analysis on the number of samples. Fourth, the fsQCA method can deal with causal asymmetry^3^ and better explain the influence of the interdependence of various condition variables on the results. Therefore, this study used the fsQCA method, and the specific analysis steps were as follows: first, we identified the outcome variable and conditional variables to be studied; second, we collected data by issuing questionnaires, deleting invalid questionnaires, and processing the data of valid questionnaires; third, the conditional variables and outcome variable of the valid questionnaires were calibrated based on recognized theoretical knowledge. The three anchor points calibrated in this study were 75% (full membership threshold), 50% (crossover point), and 25% (full non-membership threshold); fourth, we calibrated the data needed to necessitate analysis to judge which conditions must exist for the generation of outcome; fifth, we constructed a truth table. The function of the truth table was to show all possible combinations of conditional variables logically, so as to clarify the relationship between the combinations of different conditional variables and the outcome variable; sixth, conditional configuration analysis was used to explore what kind of configuration can better promote the generation of results, or what kind of configuration will inhibit the generation of results; and seventh, we conducted a robust analysis to further prove that the results were stable, scientific and applicable.

### Sample

This study conducted a questionnaire survey on university teachers in China in the form of an electronic questionnaire and e-mail. A total of 198 questionnaires were collected from participants and resulted in 164 valid questionnaires, with an effective rate of 82.83%. Of the 164 valid samples, 83 (50.61%) participants were women, and 81 (49.39%) were men; four (2.44%) participants were aged 25 and below, five (3.05%) were between the ages of 25–35 years; 62 (37.80%) were between the ages of 36–45 years, 53 (32.32%) were between ages of 46–55 years, and 40 (24.39%) participants were over 56 years old. In terms of professional titles, four (2.44%) participants did not have a grade; four (2.44%) had primary professional titles; 50 (30.49%) had intermediate professional titles; 66 (40.24%) had deputy senior professional titles; and 40 (24.39%) had senior professional titles.

### Measures

#### Big Five Personality

The Big Five personality questionnaire adopts the 10-items scale compiled by Gosling et al. (2003). The scale has 10 measurement items, of which questions 1, 3, 5, 7, and 9 are forward-scored items, and questions 2, 4, 6, 8, and 10 are reverse-scored items. The questionnaire is divided into five sections: extraversion factor (questions 1 and 6), agreeableness factor (questions 2 and 7), conscientiousness factor (questions 3 and 8), emotional stability factor (questions 4 and 9), and openness to experience factor (questions 5 and 10). Responses were provided on a five-point Likert scale (1 = “completely different” to 5 = “the same”). The Cronbach’s α of each Big Five personality trait was greater than 0.79.

#### Academic Entrepreneurial Motivation

Academic entrepreneurial motivation was based on a questionnaire prepared by Lam (2011). The scale has seven items, divided into three sections: ribbon (including funding and reputation; questions 1, 5, and 6), puzzle (including knowledge and curiosity; questions 2, 3, and 4), and gold (mainly referring to income; question 7). Items were rated on a five-point Likert scale (1 = “strongly unimportant” and 5 = “strongly important”). For the two multi-item factors, the internal consistency (Cronbach’s α) was higher than 0.88.

#### Academic Entrepreneurial Intentions

Miranda et al. (2017) prepared the academic entrepreneurial intention scale. The four items on the scale were rated on a five-point Likert scale (1 = “strongly disagree” and 5 = “strongly agree”). The Cronbach’s α of the scale was 0.81. The specific items are shown in Table 1.

**TABLE 1:** Measurement items for each variable.

Variables	Items	Authors
Big Five personality (I see myself as:)	(1) Extroverted; enthusiastic.	Gosling et al., 2003
	(2) Critical; quarrelsome.	
	(3) Dependable; self-disciplined.	
	(4) Anxious; easily upset.	
	(5) Open to new experiences; complex.	
	(6) Reserved; quiet.	
	(7) Sympathetic; warm.	
	(8) Disorganized; careless.	
	(9) Calm; emotionally stable.	
	(10) Conventional; uncreative.	
Academic entrepreneurial motivation	(1) To increase funding and other research resources.	Lam, 2011
	(2) Application and exploitation of research results.	
	(3) To create opportunities for knowledge exchange/transfer.	
	(4) To satisfy intellectual curiosity.	
	(5) To build personal and professional networks.	
	(6) To provide work placement or job opportunities for students.	
	(7) To increase personal income.	
Academic entrepreneurial intentions	(1) I am determined to create a business in the future.	Miranda et al., 2017
	(2) I intend to commercialize the results of my research through a spin-off.	
	(3) I would very much like to be an entrepreneur.	
	(4) I recently searched for information on how to create a spin-off to commercialize the results of my research.	

## Results

### Calibration of Variables

In the fsQCA method, both condition and outcome variables are considered as separate sets with different membership degrees. Uncalibrated data can only show the relative position between cases, which does not meet the set theory principle of fsQCA. Therefore, the condition and outcome variables should be calibrated before fsQCA analysis. In contrast to the implicit calibration of traditional variables (i.e., calculating the mean and standard deviation), fsQCA is mainly calibrated by external standards (direct or indirect). Since the five-point Likert scale was used in this study, the direct method was used to calibrate the variables.

Three qualitative anchor points needed to be set to calibrate variables using the direct method: full membership threshold, crossover point, and full non-membership threshold. The membership degree after calibration was between 0.0 ∼ 1.0. Based on the research of Fiss (2011), the three qualitative anchor points of eight condition variables and one outcome variable were set at 75% (full membership threshold), 50% (crossover point), and 25% (full non-membership threshold) of the sample data distribution, respectively. The specific calibration data are presented in Table 2.

**TABLE 2:** Calibration anchor points and descriptive statistics of condition variables and outcome variables.

Research variables	Variable name	Abbreviation	Fuzzy set calibration			Descriptive statistics			
			Full membership(75%)	Crossover point(50%)	Full non-membership(25%)	Mean	*SD*	Min.	Max.
Condition variables	Extraversion	EX	4.00	3.50	3.00	3.47	0.91	1.00	5.00
	Agreeableness	AG	4.00	3.50	3.00	3.57	0.83	1.00	5.00
	Conscientiousness	CO	4.50	4.00	3.00	3.89	0.85	2.00	5.00
	Emotional stability	EM	4.50	3.50	3.00	3.78	0.78	2.00	5.00
	Openness to experiences	OP	4.00	3.00	3.00	3.33	0.92	1.00	5.00
	Ribbon	RI	4.00	3.67	3.00	3.45	1.02	1.00	5.00
	Puzzle	PU	4.00	3.67	3.00	3.69	0.87	1.00	5.00
	Gold	GO	4.00	3.00	2.00	2.71	1.23	1.00	5.00
Outcome variable	Academic entrepreneurial intention	AEI	4.19	3.75	3.00	3.62	0.91	2.00	5.00

### Necessity Analysis

Necessity analysis is a necessary condition for judging whether a single variable is an outcome variable. Although the necessary condition is a condition variable that must exist to cause the result to occur, it cannot guarantee the result. Generally speaking, in fsQCA analysis, judging the necessary condition depends on whether the consistency threshold of a single condition variable is greater than 0.9. If it is greater than 0.9, the single condition variable is regarded as the necessary condition of the outcome variable. As shown in Table 3, the necessary consistency of each condition variable for high or not-high academic entrepreneurial intention was less than 0.9. Therefore, each condition variable did not constitute a necessary condition for the outcome; that is, although each condition variable had a certain degree of explanation for the outcome of academic entrepreneurial intention, its explanatory ability was relatively weak. Through necessity analysis, it was found that no single condition can lead to the necessity of high academic entrepreneurial intention. Therefore, it is necessary to further analyze the configuration of various condition variables and explore the combination path of high (or low) academic entrepreneurial intention.

**TABLE 3:** Necessity analysis.

Condition variable	AEI		∼AEI	
	Consistency	Coverage	Consistency	Coverage
EX	0.5280	0.5735	0.4627	0.5195
∼ EX	0.5576	0.5011	0.6200	0.5758
AG	0.7096	0.6420	0.4659	0.4356
∼ AG	0.3761	0.4052	0.6170	0.6871
CO	0.6489	0.6308	0.4654	0.4676
∼ CO	0.4523	0.4501	0.6325	0.6505
EM	0.6640	0.5766	0.5864	0.5263
∼ EM	0.4544	0.5153	0.5281	0.6190
OP	0.7819	0.6474	0.5141	0.4399
∼ OP	0.3236	0.3919	0.5880	0.7359
RI	0.7612	0.8044	0.2571	0.2808
∼RI	0.3194	0.2938	0.8209	0.7803
PU	0.7390	0.6769	0.4256	0.4028
∼ PU	0.3480	0.3696	0.6587	0.7228
GO	0.4636	0.5248	0.4747	0.5554
∼ GO	0.6072	0.5280	0.5938	0.5336

*(1) The wave symbol “∼” refers to “negation”; (2) “AEI” indicates the high academic entrepreneurial intention of scholars; (3) “∼AEI” indicates the not-high academic entrepreneurial intention of scholars.*

### Sufficiency Analysis of Configuration

FsQCA software was used for truth table analysis to analyze the eight condition variables (extraversion, agreeableness, conscientiousness, emotional stability, openness to experience, ribbon, puzzle, and gold) and one outcome variable (academic entrepreneurial intentions). Following the suggestions of Fiss (2011), we set the consistency threshold to 0.8 and the case frequency threshold to 1. We deleted those that did not conform to the value setting in the truth table, and further reviewed the Proportional Reduction in Inconsistency (PRI).^4^ If it was less than 0.7, the outcome variable must be encoded as a value of 0. In the necessity analysis, no single condition variable is necessary for the outcome variable. Therefore, the standardized analysis results are defaulted in standard analyses (that is, assuming that the presence or absence of each condition variable can constitute the reason for the high academic entrepreneurial intention), and three solutions are obtained: complex, parsimonious, and intermediate solutions. The complex solution is the outcome based on the original data, excluding the “logical remainder” (logically existing, but the configuration is not covered by the case), and is the solution with the largest number of configurations. That is, the complex solution does not include any analysis of counterfactual combination (in case of lack of empirical examples, it is necessary to infer whether the outcome variable is reasonable based on the combination of imaginary condition variables), and all remainder combinations are defined as “false.” The parsimonious solution includes all “logical remainder,” which is the solution with the least number of configurations, that is, the parsimonious solution includes the analysis of all counterfactual combinations (easy counterfactual and difficult counterfactual). The intermediate solution is the configuration complexity in the middle. It only considers simple counterfactual analysis and brings the “logical remainder” in line with the researcher’s theoretical and practical knowledge into the solution and cannot eliminate the necessary conditions. The function of the parsimonious and intermediate solutions is to identify the core or peripheral conditions, that is, when a condition appears in both parsimonious and intermediate solutions, the condition is the core condition; if a condition exists only in the intermediate solution, it is a peripheral condition. The core condition has a high impact on the outcome and plays a leading and promoting role, while the peripheral condition plays an auxiliary role in the outcome (Pappas et al., 2016).

As shown in Table 4, the four configurations (A1a, A1b, A1c, and A2) can be seen based on the calculation results of the fsQCA software. As the core condition (OP*RI)^5^ was the same, it constituted a second-order equivalent configuration (A1a, A1b, A1c). As shown in Table 4, the consistency of the four configurations (A1a, A1b, A1c, and A2) were 0.9494, 0.9531, 0.8839, and 0.9308, respectively, which were greater than 0.8 (theoretical value). Therefore, it was proven that the four configurations met the consistency conditions. In addition, the total solution consistency was 0.9275, which was also greater than 0.8 (theoretical value), indicating that the four configurations constituted sufficient conditions for high academic entrepreneurial intention, and the four configurations could explain the outcome. The total solution coverage was 0.4363, indicating that about 44% of high academic entrepreneurial intention could be explained by these four configurations. Furthermore, the four configurations were analyzed and described in detail.

**TABLE 4:** Configuration of high academic entrepreneurial intention.

Condition variables	Outcome variable (AEI)			
	A1			A2
	A1a	A1b	A1c	
EX		•	•	⊗
AG	•		•	⊗
CO	•	•		•
EM	•	•	⊗	•
OP	🌑	🌑	🌑	⊗
RI	🌑	🌑	🌑	🌑
PU	•	•	•	⊗
GO		⊗	•	•
Consistency	0.9494	0.9531	0.8839	0.9308
Raw coverage	0.3445	0.1662	0.1011	0.0450
Unique coverage	0.1846	0.0234	0.0476	0.0202
Solution consistency	0.9275			
Solution coverage	0.4363			

(1) The symbol of “•” indicates the presence of peripheral condition; “🌑” = the present core condition; “⊗” = the absence of peripheral condition; “⊗” = the absence of core condition; blank = the condition can or cannot appear. (2) In the standard analysis, ∼EX*RI is selected as the prime implicant.

Under the openness to experiences—ribbon—dominant path (A1, OP*RI) configuration, the three core conditions were the same; thus, A1a, A1b, and A1c constituted a second-order equivalent configuration. Among them, A1a configuration (which could explain 34% of the cases and had the largest raw coverage among the four configurations and was the most important configuration leading to high academic entrepreneurial intention) showed that regardless of whether extroverted personality traits and gold motivation exist, as long as there were two core conditions of openness to experiences and ribbon, combined with the peripheral conditions of agreeableness, conscientiousness, emotional stability, and puzzle motivation, it could guide university teachers to stimulate high academic entrepreneurial intention. The configuration of A1b showed that when gold motivation does not exist, regardless of whether there is agreeableness, scholars’ high academic entrepreneurial intention could be stimulated as long as they had the two core conditions of openness to experience and ribbon motivation, as well as certain personality characteristics of extroversion, conscientiousness, emotional stability, and puzzle motivation (peripheral condition). The configuration of A1c showed that under the personality traits without emotional stability and irrespective of the conscientiousness personality traits, as long as scholars had the two core conditions of openness to experience and ribbon motivation, as well as a certain degree of extraversion and agreeableness personality traits and a certain degree of puzzle motivation and gold motivation, it could better promote scholars’ academic entrepreneurial intention. The results from the three configurations showed that when there is a certain puzzle motivation, compared with other conditions (extraversion, agreeableness, conscientiousness, emotional stability, and gold motivation), as long as there is sufficient openness to experience personality traits and ribbon motivation, it can stimulate scholars’ high academic entrepreneurial intention. The first configuration showed that the better the openness to experience, reputational or career rewards, the more scholars can stimulate a high academic entrepreneurial intention (Vega-Gomez et al., 2020). When scholars seek innovative ideas and give play to their creativity based on academic research, especially when there are external rewards related to ribbon motivation (Lam, 2011) (such as rewarding scholars with more research funds in the form of business success or improving scholars’ academic reputation and academic professional network relations), it will further stimulate scholars’ academic entrepreneurial intention.

The ribbon—dominant path (A2, ∼EX*RI) configuration showed that when the presence of ribbon motivation and the absence of extraversion are taken as the core conditions, and combined the presence of conscientiousness, emotional stability and gold motivation, and the absence of agreeableness, openness to experience and puzzle motivation, scholars’ high academic entrepreneurial intention could be stimulated. The second configuration showed that although scholars’ do not have extraversion personality traits (i.e., they cannot actively express their self and enthusiasm in communicating with others), it does not affect the intention of scholars to start a business. As entrepreneurs, extroverts communicate more easily with others, which has a positive impact on the entrepreneurial process (Zhao and Seibert, 2006). However, scholars differ from entrepreneurs. Owing to their professional nature and habits, scholars focus more on the research content itself and do not focus as much on communicating with others and maintaining business relations (Perkmann et al., 2013). However, when there is the absence of a good, extroverted personality, it means there is the presence of analytical power and the ability to make detailed observations. If there are external motives, such as improving personal professional reputation and enhancing academic and social relations, it will inspire scholars to start a business.

### Configuration of Not-High Academic Entrepreneurial Intention

This study also examined the kind of configuration that can lead to not-high academic entrepreneurial intention, which utilizes the same measurement steps of high intention (the consistency threshold was set to 0.8, and the case frequency threshold was set to 1). Table 5 lists the results and showed four configurations that did not lead to high academic entrepreneurial intention. The total solution coverage was 29%. Among the four configurations, three configurations (E1a, E1b, and E1c) constituted the second-order equivalent configuration, that is, the core conditions of the three configurations were the same (the absence of extraversion and the absence of conscientiousness impact greatly on the configuration that does not produce high academic entrepreneurial intention).

**TABLE 5:** Configuration of not-high academic entrepreneurial intention.

Condition variables	Outcome variable (∼AEI)			
	E1			E2
	E1a	E1b	E1c	
EX	⊗	⊗	⊗	⊗
AG	⊗	⊗	⊗	⊗
CO	⊗	⊗	⊗	•
EM	⊗	⊗	⊗	•
OP		•	⊗	•
RI	⊗	⊗	⊗	⊗
PU	⊗	⊗	•	•
GO	⊗		•	⊗
Consistency	0.8673	0.8726	0.9755	0.9014
Raw coverage	0.2014	0.1488	0.0573	0.0746
Unique coverage	0.0758	0.0251	0.0158	0.0370
Solution consistency	0.8753			
Solution coverage	0.2854			

(1) The symbol “•” indicates the presence of peripheral condition; “⊗” = the absence of peripheral condition; “⊗” = the absence of a core condition; blank = the condition can or cannot appear. (2) In the standard analysis, ∼EX*∼AG*∼GO is selected as the prime implicant.

Comparing E1a and E1b, regardless of the presence of openness to experience personality traits and the absence of gold motivation peripheral conditions, and the absence of conditions (agreeableness, emotional stability, ribbon motivation, and puzzle motivation), the absence of extraversion and of conscientiousness personality traits (core conditions) led to not-high academic entrepreneurial intention. Comparing E1a and E1c, regardless of whether there was an absence of openness to experience peripheral condition and the absence or presence of puzzle and gold motivation peripheral conditions, in the absence of conditions (agreeableness, emotional stability, and ribbon motivation), the absence of extraversion and of conscientiousness personality traits led to not-high academic entrepreneurial intention. The absence of extraversion and the presence of ribbon motivation can stimulate scholars’ high academic entrepreneurial intentions (A2). However, if extraversion and conscientiousness are absent, it will not lead to high academic entrepreneurial intention. Scholars with insufficient conscientiousness will have avoidance psychology and behavior in the face of entrepreneurial failure or difficulties (Brandstaetter, 1997). Therefore, this configuration is called the extraversion—conscientiousness—inhibition path.

The configuration of E2 showed that despite the presence of conscientiousness, emotional stability, openness to experience, and puzzle motivation peripheral conditions, in the absence of extraversion, agreeableness, and gold motivation core conditions, there was no high academic entrepreneurial intention. In the absence of extraversion, agreeableness, and gold motivation, scholars are not sociable, unwilling to cooperate with others, and distrust others in the process of entrepreneurship. In addition, scholars do not have academic entrepreneurial intentions without the influence of gold motivation (D’Este and Perkmann, 2011). Good conscientiousness and agreeableness promote entrepreneurship (Zhao and Seibert, 2006). However, when these two personality traits do not exist and there is no financial motivation, scholars have no favorable conditions for academic entrepreneurship. Therefore, this configuration is called the extraversion—agreeableness—gold—hindrance path.

### Robustness Analysis

As the results of fsQCA are sensitive and random, further robustness analysis was needed to improve the scientificity and reliability of the results (Fiss, 2011). At present, there are two main types of robustness analysis methods: the set theory-specific robustness analysis and statistical theory-specific robustness analysis. As the QCA research method is based on set theory, more scholars suggest that the robustness analysis method should adopt the more suitable set theory-specific method. This study conducted two robustness analyses on the configuration results of high academic entrepreneurial intention and not-high academic entrepreneurial intention. Based on the research of Skaaning (2011), the first robustness analysis set the case threshold to 1 (unchanged) and changed the consistency threshold from 0.8 to 0.85. Finally, the configuration results of high academic entrepreneurial intention and not-high academic entrepreneurial intention were obtained, as shown in Table 6. Based on Garcia-Castro and Francoeur (2016), the second robustness analysis adjusted the full non-membership threshold of eight condition variables (extraversion, agreeableness, conscientiousness, emotional stability, openness to experience, ribbon, puzzle, and gold) and one outcome variable (academic entrepreneurial intention) from the 25th percentile to the 35th percentile, as shown in Table 7. The other conditions remained unchanged. The final configuration results of high and not-high academic entrepreneurial intentions are shown in Table 8.

**TABLE 6:** Configuration of high and not-high academic entrepreneurial intention for robustness analysis (other conditions remain unchanged, and the consistency threshold was 0.85).

Condition variables	Outcome variable AEI^➀^ (high)				Outcome variable ∼AEI^➀^ (not-high)			
	A1^➀^			A2^➀^	E1^➀^			E2^➀^
	A1a^➀^	A1b^➀^	A1c^➀^		E1a^➀^	E1b^➀^	E1c^➀^	
EX		•	•	⊗	⊗	⊗	⊗	⊗
AG	•		•	⊗	⊗	⊗	⊗	⊗
CO	•	•		•	⊗	⊗	⊗	•
EM	•	•	⊗	•	⊗	⊗	⊗	•
OP	🌑	🌑	🌑	⊗		•	⊗	•
RI	🌑	🌑	🌑	🌑	⊗	⊗	⊗	⊗
PU	•	•	•	⊗	⊗	⊗	•	•
GO		⊗	•	•	⊗		•	⊗
Consistency	0.9494	0.9531	0.8839	0.9308	0.8673	0.8726	0.9755	0.9014
Raw coverage	0.3445	0.1662	0.1011	0.0450	0.2014	0.1488	0.0573	0.0746
Unique coverage	0.1846	0.0234	0.0476	0.0202	0.0758	0.0251	0.0158	0.0370
Solution consistency	0.9275				0.8753			
Solution coverage	0.4363				0.2854			

(1) The symbol of “•” indicates the presence of peripheral condition; “🌑” indicates the present core condition; “⊗” indicates the absence of peripheral condition; “⊗” indicates the absence of core condition; blank indicates that the condition can or cannot appear. (2) In the standard analysis, the high academic entrepreneurial intention outcome chose ∼EX*RI as the prime implicant. Moreover, not-high academic entrepreneurial intention outcomes chose ∼EX*∼AG*∼GO as the prime implicant. (3) The symbol “^➀^” indicates the first robustness analysis method.

**TABLE 7:** Robustness analysis (other conditions remain unchanged, full non-membership threshold was 35%).

Research variables	Variable name	Abbreviation	Fuzzy set calibration		
			Full membership(75%)	Crossover point(50%)	Full non-membership(35%)
Condition variables	Extraversion	EX	4.00	3.50	3.00
	Agreeableness	AG	4.00	3.50	3.00
	Conscientiousness	CO	4.50	4.00	3.50
	Emotional stability	EM	4.50	3.50	3.50
	Openness to experiences	OP	4.00	3.00	3.00
	Ribbon	RI	4.00	3.67	3.00
	Puzzle	PU	4.00	3.67	3.33
	Gold	GO	4.00	3.00	2.00
Outcome variable	Academic entrepreneurial intention	AEI	4.19	3.75	3.00

**TABLE 8:** Configurations of high and not-high academic entrepreneurial intention for robustness analysis (full non-membership threshold was 35%).

Condition variables	Outcome variable AEI^➁^ (high)					Outcome variable ∼AEI^➁^ (not-high)			
	A1^➁^			A2^➁^		E1^➁^			E2^➁^
	A1a^➁^	A1b^➁^	A1c^➁^	A2a^➁^	A2b^➁^	E1a^➁^	E1b^➁^	E1c^➁^	
EX		•	•	⊗	⊗	⊗	⊗	⊗	⊗
AG	•		•	•	⊗	⊗	⊗	⊗	⊗
CO	•	•		•	•	⊗	⊗	⊗	•
EM	•	•	⊗	⊗	•	⊗	⊗	⊗	•
OP	🌑	🌑	🌑	⊗	⊗		•	⊗	•
RI	🌑	🌑	🌑	🌑	🌑	⊗	⊗	⊗	⊗
PU	🌑	🌑	🌑	⊗	⊗	⊗	⊗	•	•
GO		⊗	•	•	•	⊗		•	⊗
Consistency	0.9541	0.9604	0.8759	0.9795	0.9679	0.8558	0.8720	0.9832	0.8924
Raw coverage	0.3143	0.1414	0.0901	0.0414	0.0299	0.2014	0.1488	0.0422	0.0507
Unique coverage	0.1825	0.0216	0.0466	0.0242	0.0177	0.0789	0.0278	0.0174	0.0329
Solution consistency	0.9337					0.8672			
Solution coverage	0.4351					0.2813			

(1) The symbol of “•” indicates the presence of peripheral condition; “🌑” indicates that the present core condition; “⊗” indicates the absence of peripheral condition; “⊗” indicates the absence of the core condition; blank indicates that the condition can or cannot appear. (2) In the standard analysis, not-high academic entrepreneurial intention outcomes chose ∼EX*∼AG*∼GO and ∼ EX*∼CO*∼RI as the prime implicant. (3) The symbol “➁” indicates the second robustness analysis method.

Both robustness analyses showed that the results of this study were robust and reliable, mainly based on two aspects (Schneider and Wagemann, 2012). First, from the difference in fitting parameters, in Table 6, the total solution coverage, total solution consistency, and coverage in the robustness analysis results of high academic entrepreneurial intention had no significant change compared with Table 4. In Table 6, the total solution coverage, total solution consistency, and coverage in the robustness analysis results of not-high academic entrepreneurial intention had no significant change compared with Table 5. In addition, in Table 8, the total solution consistency of the high academic entrepreneurial intention configuration changed from 0.9275 to 0.9337, and the total solution coverage changed from 0.4363 to 0.4351; in Table 8, the total solution consistency of not-high academic entrepreneurial intention configuration changed from 0.8753 to 0.8672, and the total solution coverage changed from 0.2854 to 0.2813. Although the specific values changed slightly, there was no substantive change in the interpretation of the results.

Second, from the set relationship state, there was no change in the configuration of high academic entrepreneurial intention in Table 6 compared with Table 4 (original results of high academic entrepreneurial intention configuration). There was no change in the configuration of not-high academic entrepreneurial intention in Table 6 compared with Table 5 (original results of not-high academic entrepreneurial intention configuration). In addition, in the high academic entrepreneurial intention configuration in Table 8, solution A1a➁ was the superset of the original solution A1a (in Table 4), solution A1b➁ was the superset of the original solution A1b, solution A1c➁ was the superset of the original solution A1c, solution A2b➁ was the superset of the original solution A2, and solution A2a➁ was one more configuration than the original solution. Generally speaking, there was little change and we could substantially explain the results. Similarly, in the not-high academic entrepreneurial intention configuration in Table 8, solution E1a➁ was the superset of the original solution E1a (in Table 5), solution E1b➁ was the superset of the original solution E1b, solution E1c➁ was the superset of the original solution E1c, solution E2➁ was the superset of the original solution E2, and the changed result had no substantial change. Therefore, the initial results of this study were reliable and robust.

## Discussion and Conclusion

The current study adopted a fuzzy set qualitative comparative analysis to explore how multiple factors can affect the academic entrepreneurial intention of university scholars from two dimensions: the Big Five personality traits and academic entrepreneurial motivation. According to the results of this study, the following conclusions can be drawn. First, the results of this study showed that high academic entrepreneurial intention and not-high academic entrepreneurial intention were not affected by net effect of factors, but by the complex configuration of multiple condition variables; second, the results revealed two configurations that affect high academic entrepreneurial intention of university scholars: the openness to experience—ribbon—dominant path, and the ribbon—dominant path; third, the results also revealed two configurations for the formation of not-high academic entrepreneurial intention: extraversion—conscientiousness—inhibition and extraversion—agreeableness—gold—hindrance paths; and fourth, the study further revealed that a causal asymmetry exists between the high and the not-high academic entrepreneurial intention configurations.

This study enriches the research content of scholars’ academic entrepreneurial intention (Johnson et al., 2017; Wang et al., 2021c). In the two configurations that affect the high academic entrepreneurial intention of scholars, ribbon motivation is a jointly owned variable. As an external motivation, scholars are more willing to obtain peer recognition for academic research results, peer and social attention in the research field, and improve their influence and reputation in the field through academic entrepreneurship (Dermentzi et al., 2016; van de Burgwal et al., 2019). Furthermore, through academic entrepreneurship, scholars can obtain more resources, attract more research funds and colleague resources, and provide students with employment opportunities to promote academic research (Hayter, 2011; Ankrah et al., 2013; Beyhan and Rickne, 2015). Therefore, when scholars have strong creativity, imagination, and a thirst for knowledge, as well as sufficient ribbon motivation, they are more willing to face the challenges of the entrepreneurial process and are more willing to start academic entrepreneurship (van de Burgwal et al., 2019; Vega-Gomez et al., 2020). Alternatively, even when extraversion does not exist, if there is sufficient ribbon motivation, it can also promote scholars to conduct academic entrepreneurship. This conclusion is consistent with previous research results, in that scholars’ academic entrepreneurial intention is positively correlated with openness to experience and ribbon motivation (Zhao and Seibert, 2006; Zhao et al., 2010; Lam, 2011; Cantner et al., 2017; Vega-Gomez et al., 2020).

This study makes three main theoretical contributions to the literature. First, it provides a new perspective for studying academic entrepreneurial intention. Previous studies analyzed academic entrepreneurial intention from the “net effect” of factors. This study reveals the “joint effect” of multiple influencing factors on academic entrepreneurial intention, which is a novel research perspective. Second, this study enriches the discussion of the Big Five personality traits and academic entrepreneurial motivation in academic entrepreneurial intention. Previous studies have shown that openness to experience (Zhao and Seibert, 2006; Vega-Gomez et al., 2020) and external reputational or career rewards motivation (Lam, 2011) influence scholars’ academic entrepreneurship. The results of this study can better explain the impact of the interaction of the Big Five personality traits and key academic entrepreneurial motivation on the results of high academic entrepreneurial intention. The practical contribution of this study is twofold. From the perspective of universities, promoting teachers’ academic entrepreneurial intention can be encouraged in various ways. For example, owing to the particularity of the profession, university scholars pay more attention to academic research and do not have much interaction with others. Therefore, even if scholars do not have the personality traits of outgoing enthusiasm or engage in active interaction with others, there are still elements that schools can utilize to stimulate scholars’ academic entrepreneurial intention. Schools can choose the ribbon—dominant path. Colleges and universities should create a good and strong academic research atmosphere and further encourage scholars’ curiosity in academic research, build a communication platform between entrepreneurial models for scholars, improve the use of entrepreneurial scientific research management funds, and subject funds to stimulate the external driving force for scholars. From a personal perspective, schools can consider the openness to experiences—ribbon—dominant path. Scholars can communicate with peers who have engaged in academic entrepreneurship to increase personal, academic, and social network relationships. Through communication, opportunities for academic entrepreneurship are found. In addition, scholars should actively participate in different forms of academic exchange activities to continuously improve the openness to experience personality traits, break fixed thinking, and obtain more information and technology support to improve innovation, entrepreneurship ability, and academic reputation.

The advantage of this study is in its use of the fsQCA method to analyze the complex impact of different factors on academic entrepreneurial intention of university scholars based on configuration thinking, breaking the “net effect” paradigm in traditional research methods. However, its weakness is that the analysis of research methods was complex, and not easy to understand for those who are not familiar with fsQCA methods. In addition, this study has the following limitations that need further exploration in future research. First, this study makes a qualitative comparative analysis from the perspective of the Big Five personality traits and academic entrepreneurial motivation. Other factors that influence academic entrepreneurial intention could be studied in future research. Second, only Chinese university scholars were included in the sample for this study. Future research can collect relevant data on the academic entrepreneurial intention of university scholars in other countries to improve the universality of the research conclusions.

## Data Availability Statement

The raw data supporting the conclusions of this article will be made available by the authors, without undue reservation.

## Ethics Statement

Ethical review and approval was not required for the study on human participants in accordance with the local legislation and institutional requirements. The participants provided their written informed consent to participate in this study.

## Author Contributions

YuZ and YaZ designed the research ideas. YuZ and PW analyzed the data and wrote the initial draft of the manuscript. All authors modified the manuscript and agreed that the final version could be published.

## Conflict of Interest

The authors declare that the research was conducted in the absence of any commercial or financial relationships that could be construed as a potential conflict of interest.

## Publisher’s Note

All claims expressed in this article are solely those of the authors and do not necessarily represent those of their affiliated organizations, or those of the publisher, the editors and the reviewers. Any product that may be evaluated in this article, or claim that may be made by its manufacturer, is not guaranteed or endorsed by the publisher.

## Footnotes

1 FsQCA can convert raw data into partial membership scores between “0” and “1.”

2 Binary variable to convert the original data to “0” or “1.”

3 Causal asymmetry: it means that the reasons for the occurrence and non-occurrence of the expected result are different. For example, in this study, having openness to experience and sufficient ribbon motivation can stimulate scholars’ high academic entrepreneurial intention, but it cannot be inferred that the lack of openness to experience and ribbon motivation will lead to scholars’ low-level academic entrepreneurial intention.

4 The proportional reduction in inconsistency needs to be sorted in descending order.

5 The multiplication signs “*” indicates a logical mode of each condition variable, which generally means logical “AND.”
